# Food Behavior and Lifestyle Among Students: The Influence of the University Environment

**DOI:** 10.3390/nu17010012

**Published:** 2024-12-24

**Authors:** Calin Avram, Victoria Nyulas, Danusia Onisor, Ion Mihai Georgescu, Julianna Szakacs, Florina Ruta

**Affiliations:** 1 Department of Medical Informatics and Biostatistics, George Emil Palade University of Medicine, Pharmacy, Science and Technology of Targu Mures, Gheorghe Marinescu Street No. 38, 540136 Targu Mures, Romania; calin.avram@umfst.ro; 2 Department of Internal Medicine VII, George Emil Palade University of Medicine, Pharmacy, Science and Technology of Targu Mures, Gheorghe Marinescu Street No. 38, 540136 Targu Mures, Romania; 3 Obstetrics Gynecology 1st Department, Braila County Emergency Hospital, 810325 Braila, Romania; ion_mihai_georgescu@yahoo.com; 4 Department of Biophysics, Biotechnology, Medical and Pharmaceutical, George Emil Palade University of Medicine, Pharmacy, Science and Technology of Targu Mures, Gheorghe Marinescu Street No. 38, 540136 Targu Mures, Romania; julianna.szakacs@umfst.ro; 5 Department of Community Nutrition and Food Safety, George Emil Palade University of Medicine, Pharmacy, Science and Technology of Targu Mures, Gheorghe Marinescu Street No. 38, 540136 Targu Mures, Romania; florina.ruta@umfst.ro

**Keywords:** student lifestyle, meal planning, food behavior

## Abstract

Introduction: A key element for a healthy lifestyle in the formative years of a young adult is a balanced, healthy, individualized diet. Aim: The aim of this study was to analyze the eating behavior and lifestyles of students at a university in Romania, as well as the extent to which the study program interferes with these factors. Methods: A retrospective study was performed, with the data being collected through an online questionnaire via the Google Forms platform; data collection for the current study was carried out in November 2024 during a student session. Results: Out of the total of 501 questionnaires collected, 416 were completed by women and 85 by men. It can be observed that 57.65% of men (49 participants) eat three meals a day compared to 53.13% of women (221 participants). The university program influenced the students’ meal schedules for 59.88% (*n* = 300) of participants, while 30.94% (*n* = 155) declared that they never have an ordered meal schedule. For 39.92% of students, the exam period has an influence on the meal schedule, including an increase the intake of sweet foods (59.88%), and some declared that they forget to eat (33.93%). Conclusions: The food and lifestyle behaviors in the studied group show health risk elements, especially an increase in tobacco consumption, a lack of regular meal schedules, and the consumption of an excess of unhealthy snacks. It was found that intensive study schedules may intensify this behavior.

## 1. Introduction

Assessments of eating behavior continue to be a point of interest in research that focuses on body weight [1,2], especially since the problems of overweightness and obesity keep are maintaining an increasing trend [3,4]. Human eating behavior is complex and is influenced by a number of factors, such as gender, age, psychological, social, lifestyle, and other factors [2,5].

Although assessments of eating behavior and related factors using self-reported instruments can be subjective [6], knowing the effect of factors with the potential to influence self-reported eating behavior can be useful for developing effective strategies to prevent eating and weight disorders. Especially among young adults, the spread of nutritional diseases is astonishing, underlining the influence of social factors on our lives. The field known as the sociology of the body investigates the ways in which our bodies are affected by social influences. Our bodies are profoundly affected by our social experiences, as well as by the norms and values of the groups to which we belong. The emergence of nutritional diseases in Western societies directly coincides with the globalization of food production, which has intensified over the last 30–40 years. Teenagers around the world are at risk of nutritional diseases, rooted in feelings of shame related to their own bodies [7]. Among the dietary patterns of adolescents, some research has described the traditional, mixed pattern, composed of plant-based and animal-based foods, as healthy, while patterns that include traditional drinks, carbohydrates, and alcoholic beverages are considered unhealthy. Significantly, the nutritional risk was relatively higher among adolescents with unhealthy dietary patterns [8].

Young adults starting independent life are particularly vulnerable to developing unhealthy eating behaviors that can lead to eating disorders and/or being overweight. Some studies have explored the associations between eating behavior and age [9,10,11], physical activity [11,12], body mass index (BMI), and social factors [13,14], but few studies have looked at the interaction between the factors specific to the university environment and dietary and lifestyle behaviors among young adults, especially in Romania.

A key element for a healthy lifestyle in the formative years of a young adult is a balanced, healthy, individualized diet. In nutrition, a balanced diet is defined as the achievement of a correct ratio in terms of the intake of micro- and macronutrients necessary to support not only basic physiological functions, but also harmonious development [15]. Maintaining a balanced diet depends on eating habits that bring a benefit to the human body, such as eating balanced, regular meals, ensuring an optimal intake of liquids to maintain an optimal state of hydration, and ensuring the superior nutritional quality of the food consumed.

In the life of a young individual, there may be harmful eating habits, such as the consumption of an increased amount of salt, excessive sugar in the form of various sweets, saturated fats in the form of fast food foods, and alcohol [16], all of which should be avoided as interprandial snacks.

University education can be considered a transitional period between adolescence and adulthood, during which young adults acquire new health-related behaviors which have influences later in adult life [16,17]. Studies show that the prevalence of overweightness and obesity, weight gain, and an unhealthy lifestyle, associated with increased risk of chronic diseases, can increase during university studies [17,18,19]. University life has often been associated with poor eating habits in students. Studies in some countries have shown that the dietary intake of students was characterized by higher consumption of snacks, fast food, fried potatoes, cakes, pies, and carbonated drinks and lower consumption of fruits and vegetables [20,21,22]. Therefore, the time spent at university is recognized as an important phase for promoting health among students, with the aim of preventing the occurrence of diseases [14] by developing the tendency to study food labels in order to choose healthy foods and ingredients [23,24]. Some studies have discussed the enabling and inhibiting factors for healthy eating habits among young adults [25,26,27,28]. According to Alves and Boog [29], understanding the eating behavior of these young adults is important, because it allows for the development of health promotion interventions.

The activity schedule, consisting either of continuous teaching or alternations between teaching and breaks, plus distant locations that students need to reach in a short time, are conditions that can interfere with nutrition and encourage the development of unhealthy eating habits (e.g., eating on the run, easily accessible foods) among young students in Romania.

In this context, the purpose of this article is to analyze the eating behavior and self-reported lifestyle of students from a university in Romania. We aim to examine the extent to which the daily schedule related to the educational process and exam periods influence food intake, both quantitatively and qualitatively. Additionally, we seek to analyze lifestyle habits within a university environment.

## 2. Materials and Methods

### 2.1. Participants

The present cross-sectional study was conducted on a sample of Romanian students. The students who attended the George Emil Palade University of Medicine, Pharmacy, Science, and Technology of Târgu Mureş (UMPhST), from different faculties and years of study, were invited as participants in the study by completing an online questionnaire. Students did not receive any incentives for participating in this study. The researchers gave brief presentations during the courses to explain the purpose and importance of the study, as well as to answer students’ questions. Students were provided with detailed information about the study’s objectives, the procedures involved, and data confidentiality to assure them of the safety and relevance of their participation. The inclusion criterion was age between 19 and 30 years. The faculties where the students came from were those with a medical profile (medicine, pharmacy, dentistry, nutrition, nursing, balneophysiokinetotherapy, and medical cosmetology) and those with a science and technology profile (law, economics, letters, psychology, engineering), in undergraduate programs. For all university students, the educational process involves two semesters, with an inter-semester vacation of one week. Each semester has two modules with an interspersed session period lasting two weeks.

Of a total of 503 participants, 2 were excluded due to the incompleteness of self-reported data. Thus, data received from 501 students were analyzed.

### 2.2. Procedure

The study was conducted in accordance with the Declaration of Helsinki and approved by the Ethics Committee of the George Emil Palade University of Medicine, Pharmacy, Science and Technology of Târgu Mureș (UMPhST) on 12 November 2024; it was assigned reference number 3402. This approval was obtained for the month of November 2024, when students are in session. Potential participants were informed about the aims, scope, and organization of the study. Informed consent was obtained from all participants. Respondents completed an online questionnaire only after accepting consent. For the current study, sections on eating behavior, activity, and lifestyle were considered.

**Figure 1:**
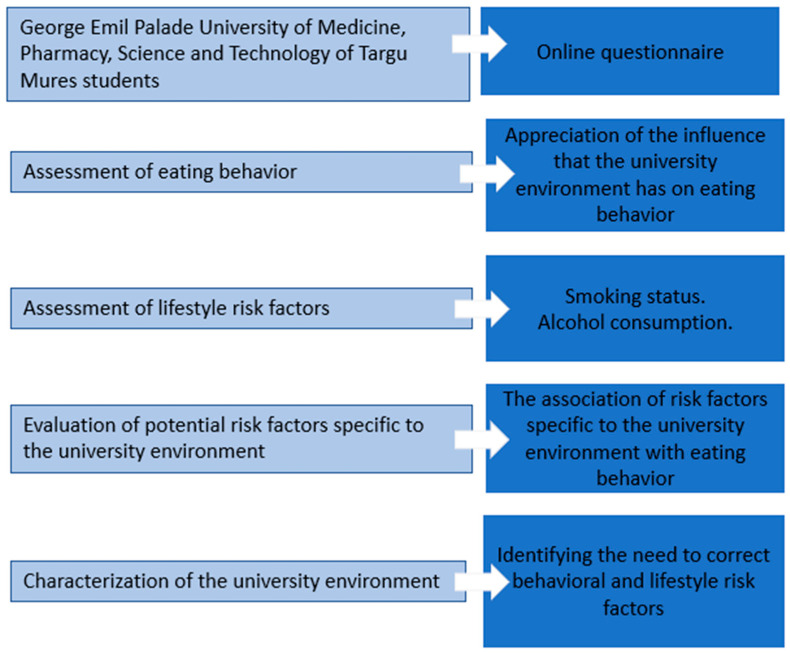
The stages of the assessment of eating behavior and risk factors in a university environment.

The exclusion criterion for the questionnaire was the city where the student carries out their university activities, as the study was conducted only in Targu Mures. The applied questionnaire consisted of 48 questions. The stages of the study are outlined in Figure 1.

The limitations of the study are due to the relatively small number of participants and the disproportionate number of women compared to men. The results of our study could be more applicable to the female student population than to men. Another limitation is the individual completion of the questionnaire by students, which may introduce biases related to the interpretation of the questions.

### 2.3. Measurements

Dietary and lifestyle behaviors were assessed using a three-factor questionnaire based on the original version of the Three-Factor Eating Questionnaire (TFEQ), developed by Stunkard and Messick [30]. The questionnaire was adapted to better identify students’ dietary lifestyle behavior. Our study used items with four possible answers, indicating the degree of agreement with the statement and scored as follows: definitely yes (3 points), rather yes (2), rather no (1), or definitely not (0) [31]. For validation, the questionnaire was distributed to a small group of students to identify any problems in terms of understanding or ambiguities.

Statistics were performed with GraphPad software version 10 (GraphPad Software, Boston, MA, USA). For variables with numerical data, we calculated the mean and SD, and for dichotomous variables, we identified their number and percentage. The chi-square test was applied to compare proportions and determine associations between variables. For numerical variables, depending on the result of the Shapiro-Wilk test [32], we applied parametric tests for unpaired data (Student *t*-test) or non-parametric tests (Mann-Whitney test). We used logistic regression to identify associations between variables of interest (e.g., gender and residency) and dietary and lifestyle behaviors. Logistic regression was chosen due to the dichotomous nature of the dependent variables (e.g., smoking, consumption of dietary supplements). The confidence threshold was set at 95% (*p* < 0.05).

## 3. Results

Out of the total of 501 questionnaires collected, 416 were completed by women and 85 by men. The average age of the participants was 23.33 (SD = 5.40), with a minimum age of 19 years and a maximum age of 30 years. The average height of the respondents was 167.46 cm (SD = 8.00).

The distribution of socio-demographic and lifestyle characteristics by gender revealed differences in physical activity, smoking status, and body weight fluctuations. It can be observed that 57.65% of men (49 participants) at three meals a day compared to 53.13% of women (221 participants) (Table 1).

More than half of the students, i.e., 53.89% (*n* = 270), ate all three main meals of the day, while 40.92% (*n* = 205) ate only two meals a day (Table 1). Furthermore, 52% of students reported eating breakfast daily, while 21% had breakfast 3–4 times a week. Finally, 69% of respondents reported eating lunch every day, and 57% reported eating dinner everyday. 

Almost half of students, 45.90% (*n* = 230), consumed food in the form of two snacks daily, 27.94% (*n* = 140) preferred a single snack, and 7.98% (*n* = 40) opted for four or several snacks a day. Sweet snacks were the most common. Chocolate was preferred by 63.87% and other sweets by 31.94%. Fresh fruits were also consumed as snacks by 59.88% and chips and other salty snacks by 47.90%.

A third of students (30.94%) never had a regular meal schedule and 23.95% usually did. The university program was found to influence students’ meal schedules for 59.88% (*n* = 300) of participants; 30.94% (*n* = 155) declared that they never have an ordered meal schedule. Most of the students, i.e., 60.88% (*n* = 305), reported that they do not consume processed food (Table 2).

The majority of young people, 31.94% (*n* = 160), consumed a sufficient amount of water daily (Table 2).

Smokers made up 30.94% of the students, while 50.50% of the students consumed alcohol very rarely and 31.33% did not consume alcohol at all (Table 1).

### Dietary and Lifestyle Behavior During the Exam Sessions

For 39.92% of students, the exam period influenced their meal schedule, leading to an increased intake of sweet foods (59.88%) and some students reported forgetting to eat (33.93%). Changes in body weight during the exam session were almost equally divided between decreases and increases (Table 2).

An increase in water intake was observed in 45.91% of students. Coffee consumption increased the most during the session.

A trend of increasing tobacco consumption during the exam session was highlighted for 26.95%, and 44.91% of the respondents confirmed that they had consumed food supplements (Table 3). Most of them (29.94%) opted for magnesium and only 15.97% for Omega 3.

The perception of the influence of exam sessions on body weight, as well as body weight fluctuations during the exam session, depended on the gender of the respondents, while smoking status was influenced by both of gender and of the environment of origin. 

The consumption of supplements was influenced by the specialization of the study program of the student (Table 4). We also found significant statistical associations between the gender of the respondents and the habit of bringing food from home, as well as the perception of optimal nutrient intake and alcohol consumption.

Skipping dinner was more frequent among medical students compared to students of other specializations (ORR = 0.1497; 95% CI 0.0295–0.7585). Male students, compared to female students, did not follow a regular schedule of three main meals per day (ORR = 3.9725; 95% CI 1.1427–13.8106). Regarding smoking, tobacco consumption was higher among males than among females (ORR = 5.2656; 95% CI 1.7015–16.2953) (Table 5).

## 4. Discussion

In our group, which consisted mainly of young female students from urban areas, mostly enrolled in the general medicine program, we observed a dietary behavior characterized by two main meals a day. Breakfast is the meal that most respondents skipped, with the majority consuming two snacks daily, preferring sweet snacks. Insufficient water intake was identified, but most respondents reported drinking enough water, with a tendency to increase consumption during exam periods. Most students stated a preference for coffee, while alcohol consumption was confirmed by a small number, with a predominant frequency of 1–2 times a week. A quarter of young people reported eating processed fast food 1–2 times a week. Weight varied both upward and downward in relatively equal percentages among students during exam periods.

One of the most important choices that young adults must make during college is choosing a healthy diet, which is key in building a stable foundation for a long-term healthy life. However, this choice seems to be difficult for students for various reasons.

Some studies have evaluated eating behaviors and physical activity by gender using principal component analysis, showing significant differences between men and women in terms of food preferences and participation in physical activities [33]. Another study examined the effects of meal frequency and meal schedules on anthropometric and metabolic outcomes, identifying the importance of meal timing and its impact on cardiometabolic health, and highlighting gender differences in responses to dietary interventions [34]. A study conducted in Sweden showed that women were more likely than men to avoid certain foods which are perceived as unhealthy, such as gluten, red meat, white flour, and food additives. Women also reported more anxiety related to diet and health [35].

Among the results obtained from a study that included students at the University of Bogota, it was noted that the time allocated for eating meals, which is strongly influenced by the schedule of courses and university activities, was the main factor in the choices made by students regarding food. Food cost and supply were also mentioned as having significance in the choices that students make. The decisions of young adults are affected by the social environment [36].

Similar results regarding changes in body weight were obtained in an international study carried out in the UK, which highlighted the poor quality of the diet of students at several British universities and its effects on young people’s weight and long-term health. As a result of this study, a correlation between unhealthy food patterns and other harmful factors on health, such as tobacco and alcohol consumption, as well as the consumption of “packaged food,” was observed. This type of diet seems to be found predominantly among young men, while female students show a preference for the “vegetarian” food model and a greater ability to cook meals at their place of residence [37].

Another study found that among the factors influencing food choices and eating behavior among college students are, taste, value for money, and accessibility. This suggests that interventions targeting the availability, accessibility, and cost of food (healthy or unhealthy) could contribute to the control of obesity among students, especially in the first years of studies [38].

Many works from the specialized literature have highlighted the association between not eating breakfast, the first main meal of the day, and the general presence of eating habits in young people’s lives, the prevalence of obesity, and even the existence of negative psychological effects on body perception. Similar values were illustrated in a 2020 study, which gathered information on the association between skipping breakfast and health problems among students from 28 countries. Among the results, the most important, in my opinion, was the decrease in academic performance [39].

A study at a university in Canada on a sample of 30 international students highlighted the change in eating habits after moving away from home. These changes were influenced by the busy study schedule, the presence of fast-food type food options “on every street corner”, and limited cooking skills [40].

Other dietary changes among students were found to be present during the exam period, highlighting excessive caffeine consumption. The intake of caffeine from drinks such as coffee and energy drinks showed a significant, alarming increase in over 60 of the surveyed students. The conclusion of a study carried out in Beirut, Lebanon, illustrated similar results, with caffeine intake during examination periods being well above the daily dose approved by the U.S. Food and Drug Administration [41].

In addition to the increase in caffeine consumption, we also observed an increase in tobacco consumption among smoking students, with a small percentage declaring no changes in their tobacco use. To combat smoking among students, thematic programs such as the Smoke-Free Medical University Week were organized. These programs aimed to raise awareness of the problems caused by smoking and to stop passive smoking among students by disseminating theoretical and practical knowledge about smoking and quitting. As a result of these actions, it was found that during the years of study, there was only a slight increase in the prevalence of active smoking, unlike in previous periods [42].

Along with these results, the obtained information also covered alcohol consumption. Alcohol consumption is not beneficial, a fact that students seem to be aware of, as 50% of alcohol consumers declared a very low intake, with only one person consuming alcohol 5–6 times a week. A low consumption of alcohol among students, similar to the previously mentioned results, was illustrated in a study conducted in Spain in 2020 [43].

The university environment can influence the eating behavior of students, most of the time in an unfavorable way [44]. In addition to the influence of the university environment, there is also the influence of the social, demographic, and eating behavior environments acquired prior to student status [45,46,47]. Students’ background, concern for body appearance, and eating habits in the context of social interactions influence eating behavior in a university environment [48,49]. Along with the components of the university environment, students’ perceptions of taste, price, and accessibility also affect eating behavior [50]. An analysis of the level of understanding among the participants regarding “healthy food” in association with their health status could contribute to the modification of eating behavior in university environments [51].

Understanding the extent to which dietary behavior impacts performance during the student period, as well as future health as adults and family health [38], could be a starting point for interventional models to implement health-promoting behavior.

The formalization of this process would allow it to be integrated within all involved factors and would enable decisions to be made much faster and more effectively to improve the quality of life of students during exam sessions [52,53].

## 5. Conclusions

The food and lifestyle behavior in the studied group showed health risk elements, especially regarding the increase in tobacco consumption associated with stressful periods specific to exam sessions. Additionally, the lack of regular meal schedules, the excessive consumption of unhealthy snacks, and intensive study schedules exacerbate these behaviors. Fluctuations in body weight can be attributed equally to the stress in periods of demanding intellectual activity and to the irregular schedules related to university curricula. The results of the present study highlight the need to address these nutritional risk factors through health education for students with the involvement of universities, implementing policies that promote healthy eating behaviors and combat sedentary lifestyles. Initially, a project to rethink university activities, which would allow easy access to hot lunches, could be prioritized.

The nutritional risk factors identified among young students are modifiable and have the potential to reduce the adoption of unhealthy patterns; these could be targeted in public health interventions. Integrated and multivariate approaches to dietary intervention are needed to promote a healthy diet and discourage the consumption of unhealthy foods, thereby reducing the tendency to adopt unhealthy dietary patterns. Improving the consumption habits regarding vegetables, seafood, and healthy local food products through pilot projects for nutrition-sensitive interventions in the study environment and beyond are strategies that should be based on the results of research in this area.

## Figures and Tables

**Table 1:** Gender distribution of study participants.

Parameters		Total (*n* = 501)	Women (*n* = 416)	Men (*n* = 85)	*p* Value
Age, Mean ± SD		23.33 ± 5.40	23.27 ± 5.61	23.58 ± 4.36	0.54 **
Height, Mean ± SD		167.46 ± 8.00	165.09 ± 5.80	179.00 ± 7.34	<0.0001 *
Weight, Mean ± SD		64.41 ± 17.34	60.43 ± 13.7	83.82 ± 20.39	<0.0001 **
Residency	Rural area	180 (35.93%)	143 (34.38%)	37 (43.53%)	0.1089 ***
	Urban area	321 (64.07%)	273 (65.63%)	48 (56.47%)	
Faculty	Medicine	341 (68.06%)	296 (71.15%)	45 (52.94%)	0.0004 ***
	Engineering	20 (3.99%)	11 (2.64%)	9 (10.59%)	
	Economics	20 (3.99%)	14 (3.37%)	6 (7.06%)	
	Letters	120 (23.95%)	95 (22.84%)	25 (29.41%)	
How many days a week are you physically active for at least 30 min?	1–2 times/week	245 (48.90%)	225 (54.09%)	20 (23.53%)	<0.0001 ***
	3–4 times/week	130 (25.95%)	120 (28.85%)	10 (11.76%)	
	5 or more times/week	85 (16.97%)	45 (10.82%)	40 (47.06%)	
	Not at all	41 (8.18%)	26 (6.25%)	15 (17.65%)	
Are you a smoker?	Yes	155 (30.94%)	125 (30.05%)	30 (35.29%)	0.3404 ***
	Not	346 (69.06%)	291 (69.95%)	55 (64.71%)	
How many main meals do you serve per day?	1 meal	10 (2.00%)	8 (1.92%)	2 (2.35%)	0.6265 ***
	2 meals	205 (40.92%)	172 (41.35%)	33 (38.82%)	
	3 meals	270 (53.89%)	221 (53.13%)	49 (57.65%)	
	4 or more meals	16 (3.19%)	15 (3.61%)	1 (1.18%)	
Do you think that the period of the examination sessions influences your eating behavior?	Yes	386 (77.05%)	336 (80.77%)	50 (58.82%)	<0.0001 ***
	Not	115 (22.95%)	80 (19.23%)	35 (41.18%)	
Do you notice weight fluctuations between term time and the rest of the academic year?	Yes	251 (50.10%)	234 (56.25%)	17 (20.00%)	<0.0001 ***
	Not	180 (35.93%)	127 (30.53%)	53 (62.35%)	
	I do not know	70 (13.97%)	55 (13.22%)	15 (17.65%)	
Do you consume dietary supplements during the session?	Yes	225 (44.91%)	190 (45.67%)	35 (41.18%)	0.4476 ***
	Not	276 (55.09%)	226 (54.33%)	50 (58.82%)	

* Unpaired *t* test, ** Mann Whitney test, *** chi square.

**Table 2:** Perceived impact on students’ diets during exam periods.

Parameters	Answer	Total (*n* = 501)	Women (*n* = 416)	Men (*n* = 85)	*p* Value
Perceived impact of the university program on students’ daily meal schedules	Definitely NO	51 (10.18%)	41 (9.86%)	10 (11.76%)	<0.0001
	Rather NO	150 (29.94%)	115 (27.64%)	35 (41.18%)	
	Rather YES	125 (24.95%)	95 (22.84%)	30 (35.29%)	
	Definitely YES	175 (34.93%)	165 (39.66%)	10 (11.76%)	
Perceived impact of the university program on students’ diet quality.	Definitely NO	61 (12.18%)	45 (10.82%)	16 (18.82%)	0.110
	Rather NO	105 (20.96%)	86 (20.67%)	19 (22.35%)	
	Rather YES	225 (44.91%)	195 (46.88%)	30 (35.29%)	
	Definitely YES	110 (21.96%)	90 (21.63%)	20 (23.53%)	
Perceived impact of exam periods (knowledge verification) on students’ eating behavior.	Definitely NO	45 (8.98%)	30 (7.21%)	15 (17.65%)	0.0004
	Rather NO	70 (13.97%)	51 (12.26%)	19 (22.35%)	
	Rather YES	216 (43.11%)	185 (44.47%)	31 (36.47%)	
	Definitely YES	170 (33.93%)	150 (36.06%)	20 (23.53%)	
Perceived impact of exam periods (knowledge verification) on students’ body weight	Definitely NO	70 (13.97%)	55 (13.22%)	15 (17.65%)	0.0065
	Rather NO	164 (32.73%)	125 (30.05%)	39 (45.88%)	
	Rather YES	155 (30.94%)	135 (32.45%)	20 (23.53%)	
	Definitely YES	112 (22.36%)	101 (24.28%)	11 (12.94%)	
Perceived impact of exam periods (knowledge verification) on students’ frequency of caloric snacks	Definitely NO	54 (10.78%)	35 (8.41%)	19 (22.35%)	0.0013
	Rather NO	80 (15.97%)	65 (15.63%)	15 (17.65%)	
	Rather YES	60 (11.98%)	50 (12.02%)	10 (11.76%)	
	Definitely YES	307 (61.28%)	266 (63.94%)	41 (48.24%)	
Perceived impact of exam periods (knowledge verification) on students’ fast food consumption	Definitely NO	50 (9.98%)	45 (10.82%)	5 (5.88%)	<0.0001
	Rather NO	79 (15.77%)	55 (13.22%)	24 (28.24%)	
	Rather YES	280 (55.89%)	255 (61.30%)	25 (29.41%)	
	Definitely YES	92 (18.36%)	61 (14.66%)	31 (36.47%)	
Perceived impact of exam periods (knowledge verification) on students’ energy drink consumption	Definitely NO	61 (12.18%)	41 (9.86%)	20 (23.53%)	<0.0001
	Rather NO	85 (16.97%)	70 (16.83%)	15 (17.65%)	
	Rather YES	219 (43.71%)	200 (48.08%)	19 (22.35%)	
	Definitely YES	136 (27.15%)	105 (25.24%)	31 (36.47%)	
Perceived impact of exam periods (knowledge verification) on students’ insufficient water consumption	Definitely NO	101 (20.16%)	95 (22.84%)	6 (7.06%)	0.0011
	Rather NO	159 (31.74%)	120 (28.85%)	39 (45.88%)	
	Rather YES	135 (26.95%)	110 (26.44%)	25 (29.41%)	
	Definitely YES	106 (21.16%)	91 (21.88%)	15 (17.65%)	
Perceived impact of exam periods (knowledge verification) on students’ increased tobacco consumption	Definitely NO	306 (61.08%)	271 (65.14%)	35 (41.18%)	<0.0001
	Rather NO	56 (11.18%)	35 (8.41%)	21 (24.71%)	
	Rather YES	67 (13.37%)	50 (12.02%)	17 (20.00%)	
	Definitely YES	72 (14.37%)	60 (14.42%)	12 (14.12%)	
Perceived impact of exam periods (knowledge check) on students’ suboptimal nutrient intake	Definitely NO	57 (11.38%)	56 (13.46%)	1 (1.18%)	<0.0001
	Rather NO	46 (9.18%)	40 (9.62%)	6 (7.06%)	
	Rather YES	294 (58.68%)	255 (61.30%)	39 (45.88%)	
	Definitely YES	104 (20.76%)	65 (15.63%)	39 (45.88%)	
Do you have a fixed meal schedule	Definitely NO	154 (30.74%)	140 (33.65%)	14 (16.47%)	0.0002
	Rather NO	210 (41.92%)	160 (38.46%)	50 (58.82%)	
	Rather YES	120 (23.95%)	105 (25.24%)	15 (17.65%)	
	Definitely YES	17 (3.39%)	11 (2.64%)	6 (7.06%)	
To what extent do the university’s programs consider their impact on the meal schedule	Definitely NO	50 (9.98%)	40 (9.62%)	10 (11.76%)	<0.0001
	Rather NO	149 (29.74%)	115 (27.64%)	34 (40.00%)	
	Rather YES	126 (25.15%)	95 (22.84%)	31 (36.47%)	
	Definitely YES	176 (35.13%)	166 (39.90%)	10 (11.76%)	

**Table 3:** Identifying associations between gender and residence with indicators of a healthy diet.

Parameters	Women (*n* = 416) vs. Men (*n* = 85)	Urban Area (*n* = 321) vs. Rural Area (*n* = 180)
Main meals served per day	0.774	0.206
Frequency of serving breakfast	0.158	0.356
Frequency of serving lunch	0.659	0.617
Frequency of serving dinner	0.044 *	0.496
Number of snacks served daily	0.987	0.680
Sweet snacks, chocolates and other sweets	0.061	0.674
Salty snacks and crisps	0.343	0.610
Fruit and vegetable snacks	0.660	0.248
Adherence to a fixed meal schedule	0.491	0.102
The influence of student activity on the meal program	0.281	0.734
Frequency of fast-food consumption	0.536	0.709
Water consumption	0.603	0.591
Physical activity of at least 30 min	0.001 *	0.222
Consume alcoholic beverages	0.426	0.403
Tobacco consumption during the session	0.038 *	0.066

* *p* < 0.05.

**Table 4:** Identifying associations among the type of faculty, gender, and residency with lifestyle during the study session.

Variabiles	Medicine Faculty (*n* = 341) vs. Others Faculty (*n* = 160)	Women (*n* = 416) vs. Men (*n* = 85)	Urban Area (*n* = 321) vs. Rural Area (*n* = 180)
Do you think that the period of the examination sessions influences your eating behavior?	0.207	0.063 *	0.461
Do you notice weight fluctuations between the session period and the rest of the academic year?	0.053	0.006 *	0.582
Weight gain/loss during the session	0.015 *	0.01 *	0.999
Do you think that your water intake changes during the session?	0.256	0.601	0.591
Coffee consumption during the session	0.826	0.789	0.669
Have you noticed the increasing trend of tobacco consumption instead of food consumption during the session?	0.779	0.038 *	0.066
During the onsite university activity, do you take a bottle of water with you?	0.549	0.013 *	0.823
Do you consume food supplements during the session?	0.046 *	0.794	0.678
During the onsite university activity, do you take packed food from home with you?	0.099	0.001 *	0.949
During the onsite university activity, do you frequent the university canteen?	0.348	0.609	0.816
From a qualitative point of view, do you think you have an optimal intake of nutrients?	0.923	0.010 *	0.101
How often do you consume alcoholic beverages?	0.125	0.004 *	0.623

* *p* < 0.05.

**Table 5:** Identifying associations among the type of faculty and gender with irregular meal schedule during the study session.

	Medicine Faculty (*n* = 341) vs. Others Faculty (*n* = 160)		Women (*n* = 416) vs. Men (*n* = 85)	
	Odds Ratio	95% CI	Odds Ratio	95% CI
Skipping breakfast	3.8698	0.8013–18.6901	1.1609	0.3528–3.8197
Skipping lunch	0.5866	0.1130–3.0446	0.5382	0.1649–1.7569
Skipping dinner	0.1497	0.0295–0.7585	0.4884	0.1409–1.6928
Less than three main meals a day	0.7520	0.1524–3.7094	3.9725	1.1427–13.8106
More than two snacks a day	0.4459	0.1022–1.9463	1.4367	0.4876–4.2329
Breakfast outside the house	1.6549	0.3321–8.2456	0.4254	0.1135–1.5952
Lunch outside the house	0.7370	0.1919–2.8310	1.4821	0.5166–4.2518
Dinner outside the house	4.6981	0.7497–29.4426	0.4179	0.0896–1.9488
Smoking	1.3894	0.3699–5.2189	5.2656	1.7015–16.2953
Alcohol consumption	1.2725	0.3643–4.4445	0.3881	0.1373–1.0967

## Data Availability

The data that support the findings of this study are available from the corresponding author upon reasonable request.
